# Utilizing induced pluripotent stem cells (iPSCs) to understand the actions of estrogens in human neurons

**DOI:** 10.1016/j.yhbeh.2015.06.014

**Published:** 2015-08

**Authors:** Carole Shum, Sara C. Macedo, Katherine Warre-Cornish, Graham Cocks, Jack Price, Deepak P. Srivastava

**Affiliations:** a Department of Basic and Clinical Neuroscience, Cell and Behaviour Unit, The James Black Centre, Institute of Psychiatry, Psychology and Neuroscience, King's College London, London SE5 8AF, UK; b Faculty of Engineering, Universidade do Porto, 4200-465 Porto, Portugal

**Keywords:** Stem cells, 17β-estradiol, Estrogen receptor, Synapse, Dendrite, Dendritic spines, Psychiatric, Schizophrenia, Autism spectrum disorders, Neurodevelopmental disorders, Neurodegenerative disease

## Abstract

This article is part of a Special Issue “Estradiol and Cognition”.

Over recent years tremendous progress has been made towards understanding the molecular and cellular mechanism by which estrogens exert enhancing effects on cognition, and how they act as a neuroprotective or neurotrophic agent in disease. Currently, much of this work has been carried out in animal models with only a limited number of studies using native human tissue or cells. Recent advances in stem cell technology now make it possible to reprogram somatic cells from humans into induced pluripotent stem cells (iPSCs), which can subsequently be differentiated into neurons of specific lineages. Importantly, the reprogramming of cells allows for the generation of iPSCs that retain the genetic “makeup” of the donor. Therefore, it is possible to generate iPSC-derived neurons from patients diagnosed with specific diseases, that harbor the complex genetic background associated with the disorder. Here, we review the iPSC technology and how it's currently being used to model neural development and neurological diseases. Furthermore, we explore whether this cellular system could be used to understand the role of estrogens in human neurons, and present preliminary data in support of this. We further suggest that the use of iPSC technology offers a novel system to not only further understand estrogens' effects in human cells, but also to investigate the mechanism by which estrogens are beneficial in disease. Developing a greater understanding of these mechanisms in native human cells will also aid in the development of safer and more effective estrogen-based therapeutics.

## Highlights

• Advances in stem cell biology allow for the reprogramming of somatic cells into induced pluripotent stem cells (iPSCs).
• Human iPSCs (hiPSCs) can be differentiated into defined neuronal lineages.
• hiPSCs can be used to investigate basic mechanisms of neural development.
• Reprogramming of somatic cells from patients has allowed investigations into complex disorders of the CNS.
• Preliminary data indicates that hiPSCs are responsive to 17β-estradiol.

## Introduction

There are multiple lines of evidence that estrogens exert a powerful influence over cognition (Brinton, 2009; Daniel, 2013; Frick, 2012; Galea et al., 2008; Luine, 2008, 2014). Studies using animal models have demonstrated that estrogens, in particular 17β-estradiol, can influence hippocampal and cortical brain regions to modulate cognitive function, including learning and memory (Frick, 2009; Galea et al., 2008; Luine, 2008). This is in addition to the effects 17β-estradiol has on reproductive and sexual behaviours, regulated by its actions in the hypothalamus (Micevych et al., 2009; Roepke et al., 2011). At the cellular levels, the effects on cognitive function are thought to be driven by 17β-estradiol's effects on synapse structure and function (Brinton, 2009; Srivastava et al., 2013). In addition to these neurotrophic effects, multiple studies have also indicated that 17β-estradiol has potent neuroprotective actions (Arevalo et al., 2015), and has been suggested to be a possible therapeutic avenue for the treatment of several neurodevelopmental, psychiatric and neurodegenerative disorders (Gillies and McArthur, 2010; Hughes et al., 2009; Srivastava and Penzes, 2011).

To date, much of our understanding of the molecular and cellular mechanisms that underlie the effect of estrogen have come from animal based in vitro and in vivo models. Conversely, our understanding of the mechanisms that underlie estrogens' effects in human neurons is limited. Indeed, it has not been possible to investigate the actions of estrogens at a molecular level in native human neurons, and thus to validate whether or not the actions of estrogens as determined in animal models are comparable to its actions in human neurons. This is in part due to the availability of, and the ethical considerations when using human tissue. These issues result in the lack of a suitable and reproducible cellular system that faithfully recapitulates a human neuronal cellular environment and that allows detailed cellular and molecular studies to be carried out. It is also important to recognise that while animal studies support a beneficial role for estrogens in a range of neurodevelopmental, psychiatric and neurodegenerative disorders, how these data translates to humans is unclear. This is particularly important, when considering that there has not been much success in translating preclinical work into novel therapeutic agents to treat debilitating neurological, neurodevelopmental or neurodegenerative disorders. This lack of conversion is due to many factors, but are likely to include species differences, differences in brain complexity and disease-specific phenotypes (Dragunow, 2008). Another important factor to consider is the potential negative effects of estrogen, or estrogen-based therapies such as increased risk of cardiovascular problems and increased risk of developing cancers. An alternative approach would be to mimic estrogenic-mediated positive effects by modulating specific ERs (Hughes et al., 2009; Zhao et al., 2005) and/or regulating 17β-estradiol intracellular molecular targets. Such strategies could exploit the beneficial effects of estrogens without the harmful side effects. Therefore, in order to utilize estrogens or estrogen-based therapeutic for the treatment of neurodevelopmental or neurodegenerative disorders, a greater understanding of the effects these compounds have on native human cells and in a disease context is critical (Gillies and McArthur, 2010; Hughes et al., 2009; Srivastava and Penzes, 2011).

Recent advances in stem cell biology are now providing us the tools in which to study basic and disease mechanisms in native human neurons (Brennand et al., 2012; Cocks et al., 2014; Dolmetsch and Geschwind, 2011; Gaspard and Vanderhaeghen, 2011). This has led to the ability to reprogram patient somatic cells into human induced pluripotent stem cells (hiPSCs) and the subsequent differentiation into neurons of specific lineages (Dolmetsch and Geschwind, 2011; Gaspard and Vanderhaeghen, 2011). Importantly, these cells encapsulate and recapitulate the genetic landscape and cellular abnormalities associated with complex disease (Durak and Tsai, 2014; Yu et al., 2013). Critically, this approach provides a potentially limitless source of live human cells for understanding basic neurobiology and disease pathophysiology, and for modelling the actions of potential drug targets (Brennand et al., 2012; Cocks et al., 2014; Dolmetsch and Geschwind, 2011; Gaspard and Vanderhaeghen, 2011). In this review, we will review a) the evidence that estrogens influence human cognition and maybe beneficial in the treatment of neurodevelopment/psychiatric disorders; b) recent advances in our ability to generate hiPSCs and their use in elucidating both basic and disease relevant mechanisms; c) the current limitations and efforts to overcome them when using iPSCs; and d) present some preliminary data demonstrating that neurons differentiated from hiPSCs are responsive to 17β-estradiol treatment.

### How do estrogens influence cognition?

During early brain development 17β-estradiol has many roles, ranging from the control of cell proliferation and apoptosis to synaptogenesis and neurogenesis (McCarthy, 2008; Sakuma, 2009). In addition, 17β-estradiol is a critical factor in determining sexual differentiation during development. It has an organisational role which contributes to the establishment of sex differences by influencing the sexually dimorphic formation of the neural circuitry that encodes reproductive and socio-aggressive behaviours (H. Lee et al., 2014; Ubuka and Tsutsui, 2014; Unger et al., 2015; Yang and Shah, 2014). Accumulating evidence indicates that 17β-estradiol's ability to regulate synapse structure and function, and thus neural circuitry, underlies its influence over cognitive function (Luine and Frankfurt, 2012; Sellers et al., 2014; Srivastava et al., 2013). In the cortex and hippocampus, 17β-estradiol has been shown to modulate dendritic spine and synapse formation and density (Luine and Frankfurt, 2012; Srivastava et al., 2013), long-term potentiation (LTP) (Foy et al., 1999; Kramar et al., 2009; Xiao et al., 2012) and long-term depression (LTD) (Mukai et al., 2007). Indeed, regulation of these cellular parameters are thought to be key events and cellular correlates of memory and learning (Fu and Zuo, 2011; Holtmaat and Svoboda, 2009; Malenka and Bear, 2004; Morris, 2003).

The actions of 17β-estradiol are mediated by the classic estrogen receptors (ERs) ERα, ERβ, as well as the G-protein coupled receptor, GPER1 (Brinton, 2009; Sellers et al., 2014; Srivastava and Evans, 2013). These receptors mediate both rapid, membrane-initiated signalling and longer-term/chronic actions via the regulation of gene transcription (Brinton, 2009; McCarthy, 2008; Srivastava et al., 2013). Both ERα and ERβ dimerize in response to 17β-estradiol binding, and subsequently translocate to the nucleus, where they can bind and influence the expression of certain genes (Greene et al., 1986). However, there is a growing appreciation that 17β-estradiol can act via ERα, ERβ and GPER1 to rapidly regulate non-classical signalling resulting in a modulation of cellular physiology (Spencer et al., 2008; Srivastava et al., 2013; Woolley, 2007). Activation of these non-classical pathways by 17β-estradiol can result in multiple cellular effects, including immediate effects on cell physiology and even on protein synthesis or gene transcription (Sellers et al., 2014). Importantly, signalling via specific ERs and the activation of these pathways have also been shown to be required for 17β-estradiol-mediated enhancements of cognitive function (Ervin et al., 2013; Frick, 2012; Gabor et al., 2015; Hawley et al., 2014; Luine and Frankfurt, 2012; Srivastava et al., 2013). It is also important to note that the precise establishment of neural circuitry during development, as well as the proper regulation and maintenance of synaptic connectivity throughout the lifetime of an animal, is essential for normal brain/cognitive function. Indeed disruptions in these process are thought to be a major contributing factor to a number of neurodevelopmental and neurodegenerative disorders (Penzes et al., 2011; Tau and Peterson, 2010; van Spronsen and Hoogenraad, 2010). As such, the ability of 17β-estradiol to regulate synapse structure and function may contribute to its beneficial effects in disease (Srivastava and Penzes, 2011; Srivastava et al., 2013).

While the effects of estrogens on cognition have been well established in animal models, the reported effects of estrogens on cognitive function in human have been much more varied (Luine, 2014; Sherwin, 2012). Nevertheless, multiple studies in human females have reported that administration of estrogens have a positive effect on cognitive function, including memory (Duff and Hampson, 2000; Hampson and Morley, 2013; Hogervorst and Bandelow, 2010; Sherwin, 2012; Smith et al., 2006). In addition, several studies have suggested that 17β-estradiol levels correlate with cognitive performance. For example, during the midluteal phase when 17β-estradiol levels are at their relative height, women have been shown to have a transient increase in performance in typically female-favouring measures of cognition such as verbal fluency. This is opposed to the menstrual phase in these same women, during which 17β-estradiol decline correlates with a transient increase in performance in typically male-favouring measures of cognition such as spatial ability (Hampson, 1990). This relationship between estrogen concentration and cognition has since been reiterated by several studies (Hampson and Morley, 2013; Hogervorst et al., 2004; Phillips and Sherwin, 1992b). In addition, the loss of estrogens (and other steroids) following menopause has been suggested to dramatically increase a woman's risk of memory loss (Maki and Henderson, 2012; Ryan et al., 2012). Interestingly, it has also been shown that this decline can be attenuated by administering exogenous estrogens relatively early in menopause (Phillips and Sherwin, 1992a; Sherwin, 1988). However not all studies have reported positive effects on cognition, with studies reporting no or even negative effects (Daniel, 2013; Hogervorst and Bandelow, 2010; Luine, 2014; Sherwin, 2012). As discussed by Luine (2014) in the primer for this special issue, the variation seen in human studies could be due to difficulties in experimental design or potential environmental cofounders. However, another possibility is that estrogens do not affect human cognition in the same manner as that seen in animal models, due to differences in the basic underlying molecular and cellular mechanisms.

### Estrogens and disease: therapeutic potential?

There is also substantial evidence that estrogens exert neuroprotective effects and may also have beneficial effects in animal models of disease (Arevalo et al., 2015; Frick, 2012; Gillies and McArthur, 2010). Preclinical studies have provided evidence that estrogen, or estrogen-based approaches are neuroprotective and could be used in the treatment of neurodevelopmental and neurodegenerative disorders such as schizophrenia (Gogos and van den Buuse, 2015; Labouesse et al., 2015), depression (Bredemann and McMahon, 2014; Hajszan et al., 2010; Walf et al., 2008), Parkinson's disease (Bourque et al., 2012; Rodriguez-Perez et al., 2013) and Alzheimer's disease (Logan et al., 2011; Zhang et al., 2012; Zhao et al., 2013). It has also been hypothesized that the beneficial effects of 17β-estradiol in these disorders are mediated, in part, through the modulation of neural circuitry (Frick, 2012; Gillies and McArthur, 2010; Hughes et al., 2009; Srivastava and Penzes, 2011). For example, the antidepressive effect of 17β-estradiol in a learned helplessness model of depression occurs concurrently with an increase in spinogenesis and LTP in CA1 neurons (Bredemann and McMahon, 2014; Hajszan et al., 2010). Furthermore, selective activation of ERβ has anti-depressive-like effects in a number of cognitive tests (Walf et al., 2008); this is in addition to ERβ-mediated modulation of synapse structure and function (Kramar et al., 2009; Srivastava et al., 2010). Interestingly, Logan et al. (2011), demonstrated that 17β-estradiol was sufficient to rescue deficits in dendritic spine density induced by soluble beta amyloid (Aβ) oligomers in neuronal cultures. Moreover, the authors reported that administration of 17β-estradiol prevented Aβ oligomer-induced impairment of inhibitory avoidance tasks (Logan et al., 2011), indicating that 17β-estradiol's regulation of synapse structure contributes to its beneficial effects on Aβ-induced cognitive deficits.

Several clinical studies and meta-analyses have been carried out investigating the potential beneficial roles of 17β-estradiol or selective estrogen receptor modulators (SERMs) in a range of disorders including schizophrenia (Kindler et al., 2015; Kulkarni et al., 2015; Torrey and Davis, 2012; Weickert et al., 2015), major depression (Kornstein et al., 2010; Young et al., 2007), and even Alzheimer's disease (Maki, 2013; Wharton et al., 2011). For example, in a recent large-scale, randomized-control study, Kulkarni et al. (2015) reported that administration of 17β-estradiol to treatment-resistant female schizophrenic patients, resulted in a clinically relevant amelioration of schizophrenic symptoms. In this study, 200 μg of 17β-estradiol was delivered via a transdermal patch to female patients for 8 weeks; patients receiving this treatment showed a greater decrease in the positive and negative syndrome scale (PANSS) than 100 μg 17β-estradiol or placebo. Several recent studies have also investigated the therapeutic potential of the SERM raloxifene in schizophrenia. In a 12 week double-blind, placebo-controlled study, orally administered raloxifene improved probabilistic association learning and significantly increased fMRI blood oxygen level-dependent (BOLD) activity in the hippocampus and parahippocampal gyrus relative to placebo, in male and female schizophrenic patients (Kindler et al., 2015). In a subsequent study by the same group, raloxifene, administered orally, improved memory and attention/processing speeds in male and female schizophrenic patients, compared to placebo (Weickert et al., 2015).

While these studies do support a positive role for estrogens, or estrogen-based therapies (Craig and Murphy, 2010; Cyr et al., 2000; Frick, 2009; Gillies and McArthur, 2010; Kulkarni et al., 2008; J.H. Lee et al., 2014; Maki and Henderson, 2012; Sherwin, 2009; Torrey and Davis, 2012), they do not support estrogens as a long-term treatment. Previous studies from the Women's Health Initiative (WHI) investigated the potential of Hormone Replacement Therapy (HRT) as a therapeutic avenue for the treatment of ageing and dementia. The findings of the WHI studies reported a decrease in cognitive function and an increased risk of dementia and stroke in women over 65 years of age who received conjugated equine estrogens (CEE) plus medroxyprogesterone acetate (MPA) compared to those who received placebo (Espeland et al., 2004; Rossouw et al., 2002; Shumaker et al., 2004). However, these studies were carried out in females that were postmenopasual for ~ 15–20 years, and the HRT studies used horse derived estrogens, made up mostly of estrone, an estrogen that has varied and often opposing effects to 17β-estradiol, and progesterone on cognition in animal models (Barha et al., 2010; Barha and Galea, 2013; Engler-Chiurazzi et al., 2012; McClure et al., 2013). Moreover, a direct comparison of CEE and 17β-estradiol on verbal memory performance in postmenopausal women indicates that 17β-estradiol has a more potent effect on memory than CEE (Wroolie et al., 2011). It is also thought that the physiological status of women is critical in determining the effectiveness of estrogen on cognition and it has been hypothesized that postmenopausal women lose their responsiveness to estrogens about 5 years after menopause (Asthana et al., 2009; Craig et al., 2005; Hogervorst and Bandelow, 2010; Maki, 2013; Maki and Henderson, 2012; Singh et al., 2013). Indeed, basic studies have also hypothesized that there is a critical period, or “window of opportunity” following menopause or surgical removal of ovaries, when the brain is still responsive to estrogens and the hormone can exert positive effects (Singh et al., 2013; Vedder et al., 2014). Conversely, treatment with estrogens after this time may exert negative, or adverse, effects on cognition (Asthana et al., 2009; Maki, 2013; Sherwin, 2009).

As discussed above, our understanding of the potential beneficial effects of estrogens, or in developing novel estrogen-based therapies, is limited due to the inability to perform in depth molecular studies of estrogens in human neurons that faithfully recapitulate the genetic, and therefore the cellular environment of specific diseases. Understanding such mechanisms would not only enhance our understandings of estrogen function in humans, but would enable us to develop safer and more effective estrogen-based therapies. Other alternatives, such as human post-mortem brain tissue and genetically-modified model organisms have provided insights into how estrogens are beneficial in a number of disorders, but these approaches have limitations. Post-mortem tissue is not living tissue and does not allow researchers to investigate the progression of the disorder. A limitation of animal models is that they often do not faithfully reflect human pathophysiology. Moreover, many disorders of the brain have a complex genetic underpinning, and thus it is not currently possible to fully recapitulate the complex genetic landscape in traditional animal models. Therefore, the ability to determine the potential effectiveness of therapeutic agents, such as estrogens, or to identify and test novel estrogen-based compounds/therapies is currently limited. If we are to fully recognise the potential of estrogen-based therapies, whether it be for females or for males, it is critical to investigate them in a cellular model which encapsulates the complex nature of these diseases, and within a native human cellular environment.

## Generation and differentiation of hiPSCs

### iPSC reprogramming technology

The method of reprogramming adult somatic cells to pluripotent stem cells was first described in 2006 by Takahashi and Yamanaka. They reported that dermal fibroblasts from adult mice could be reprogrammed into a pluripotent state by retroviral transduction of four transcription factors: *OCT4*, *KLF4*, *c-MYC* and *SOX2* (Takahashi and Yamanaka, 2006). The reprogrammed cells were termed induced pluripotent stem cells (iPSCs), and are similar to embryonic stem cells (ESCs) in their morphology, proliferation, surface antigens, gene expression and capacity to differentiate into the cell types of the three primordial germ layers. A year later, Takahashi et al. (Takahashi et al., 2007b) applied the same technology to human adult dermal fibroblasts to generate the first human iPSCs (hiPSCs). Yamanaka's seminal studies provided an avenue to generate patient and disease-specific iPSCs and led to his being awarded the Nobel Prize in Medicine and Physiology in 2012. This discovery, combined with protocols for the directed differentiation of neurons, enabled access to these cell types without the ethical issues involved with the use of human embryonic stem cells.

Since this discovery, many others have shown that it is possible to generate hiPSCs from other adult somatic cell types, including peripheral blood (Loh et al., 2009), hair follicles (Aasen et al., 2008), amniotic cells (Li et al., 2009; Zhao et al., 2010), cells present in urine (Zhou et al., 2012) and various other cell types (Aoki et al., 2010; Bar-Nur et al., 2011; Eminli et al., 2009; Giorgetti et al., 2009; Haase et al., 2009; J.B. Kim et al., 2009; Liu et al., 2010; Nakagawa et al., 2008; Sugii et al., 2011; Yu et al., 2007). Although a well-established cell type in many fields of research, due to their ease of handling and the cost-effectiveness, there are disadvantages to the use of fibroblasts as a starting cell type for producing hiPSCs. Patient dermal fibroblasts are obtained from painful skin punch biopsies that present risk of infections and allergic reactions to anaesthetics, and must be performed by trained professionals. In addition, fibroblasts are reprogrammed with a longer time frame and less efficiency than other somatic cell types (Aasen et al., 2008). Thus, these studies have advanced hiPSC research by enabling non-invasive methods of acquiring starting material and reducing the time and costs, while increasing the efficiency of reprogramming.

**Fig. 1:**
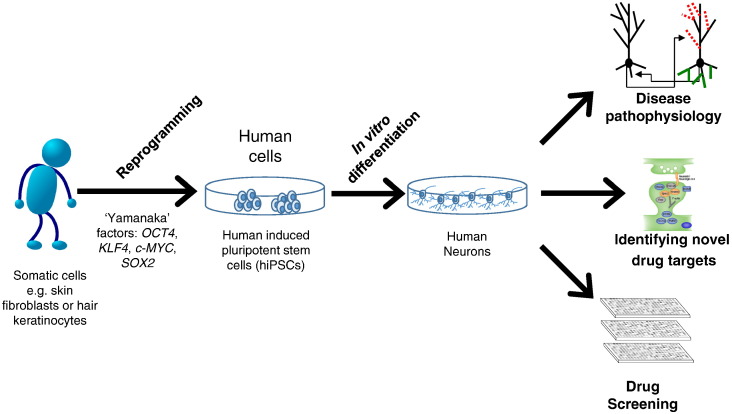
**Promise of hiPSCs.** Schematic representation of how somatic cells taken from a patient can be reprogrammed into induced pluripotent stem cells (iPSCs) using the ‘Yamanaka’ factors, *OCT4*, *KLF4*, *c-MYC* and *SOX2*. Subsequent differentiation of human iPSCs (hiPSCs) into neurons of define lineage allow for investigations into disease pathophysiology and identification of potential drug targets. In addition, hiPSC derived neurons may function as a cellular platform in which drug screens can be carried out using disease relevant neurons.

Conventional hiPSC reprogramming has made use of integrating viral vectors, such as retroviral and lentiviral vectors, for the delivery of the four pluripotency factors (*OCT4*, *KLF4*, *c-MYC* and *SOX2*) into the starting cell types (Takahashi et al., 2007b; Yu et al., 2007). Integrating viral vectors were critical in the development of iPSC technology due to its ability to enable long-term transgene expression, but result in the integration of viral DNA into the host genome. This type of transgene delivery has disadvantages, such as the risk of insertional mutagenesis, residual expression of integrated transgenes, and uncontrolled activation or inactivation of the integrated transgenes, which is critical in the case of iPSC reprogramming, since all four of the pluripotency factors are oncogenic (Hu, 2014). Tremendous effort has since led to the development of alternative protocols to avoid the integration of the pluripotency factors into the host genome. It is now possible to generate hiPSCs with the use of episomal vectors (Sendai vectors; (Fusaki et al., 2009)), non-integrating viral vectors (Sarkis et al., 2008), small molecules (Ichida, 2009; Lyssiotis et al., 2009; Shi et al., 2008), protein transduction (D. Kim et al., 2009) and microRNAs (Pfaff et al., 2011; Subramanyam et al., 2011) (Fig. 1). These methods have addressed many of the issues associated with integrating viral vectors and advanced hiPSC research by producing hiPSC lines with increased efficiency, low toxicity and free of transgene footprints, making them feasible for clinical studies such as cell replacement therapies. The efficiency of hiPSC generation has greatly improved since Takahashi and Yamanaka's initial breakthrough, and the technology continues to develop.

### Neuronal differentiation of hiPSCs

A key component of using hiPSCs to elucidate basic and disease relevant mechanisms is the ability to direct the differentiation of stem cells to specific neuronal cell types. The pathways involved in neural development were first elucidated from studies of animal embryology. The first step in the development of the neural tube, neural induction, is believed to be the default pathway, involving the bone morphogenetic proteins (BMPs), Wnt and fibroblast growth factor (FGF) signalling pathways (Aubert et al., 2002; Chambers et al., 2009; LaVaute et al., 2009). Neural induction leads to a default and primitive anterior identity, which is subsequently patterned by extrinsic morphogens such as Wnts, FGFs, retinoic acid and Sonic Hedgehog (Shh), to give rise forebrain, midbrain, hindbrain or spinal cord domains.

**Fig. 2:**
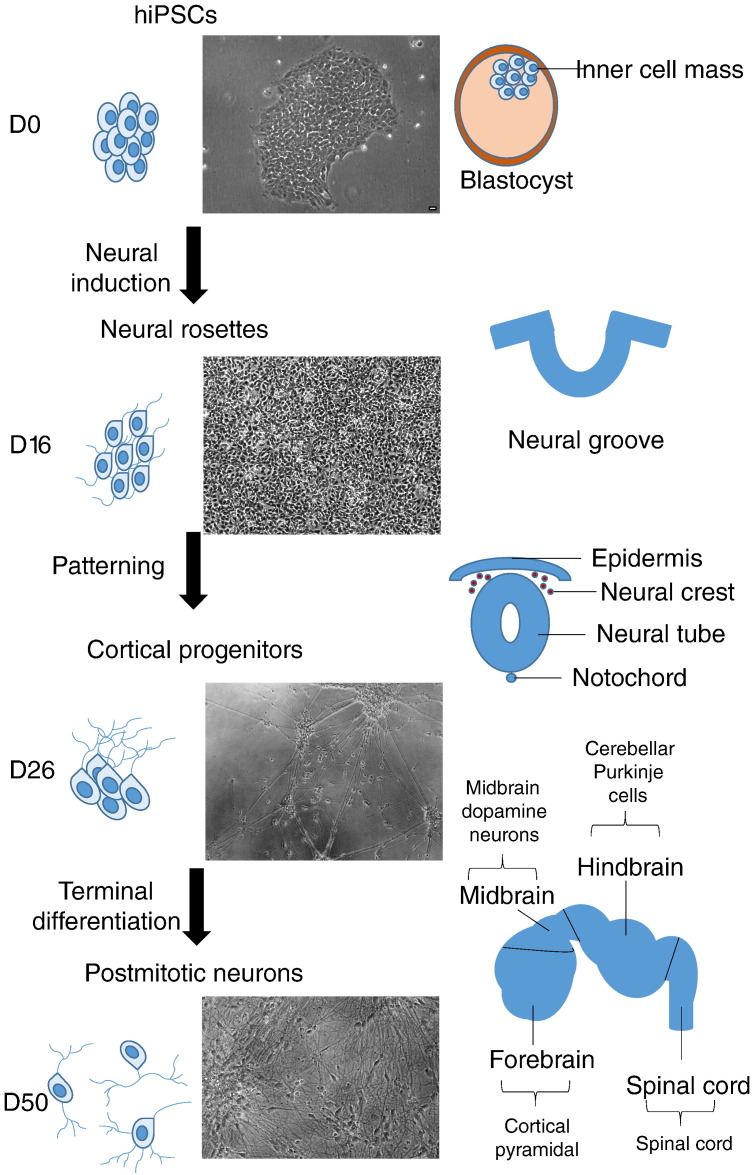
**Schematic representation of in vitro neural differentiation and human neural development.** Schematic representation of the mechanisms of in vitro neural differentiation of hiPSCs and in vivo neural induction and early patterning of the neural plate/tube. hiPSCs neural induction and neural progenitor specification follow processes as in vivo to give rise to well-defined neuronal populations. Before neural induction, hiPSCs correspond to pluripotent cells within the inner cell mass of the blastocyst. Neural induction, regulated by bone morphogenetic proteins (BMP), Wnt and fibroblast growth factors, gives rise to neuroprogenitor cells (NPCs), and the formation of neural rosette structures, which recapitulates a default anterior identity. Subsequent patterning by addition of extrinsic morphogens including Wnts, FGFs, retinoic acid and Sonic Hedgehog, give rise to forebrain, midbrain, hindbrain or spinal cord progenitors and subsequently post-mitotic neurons. Scale bar = 10 μm.

Neuronal differentiation of hiPSCs follow the same pathways as in vivo to give rise to mature neuronal populations (Shi et al., 2012) (Fig. 2). The most efficient neural induction of hiPSCs is achieved by dual inhibition of the SMAD signalling pathway (Chambers et al., 2009). This involves the synergistic inhibition of the BMP and TGFβ pathways to achieve rapid and uniform neural conversion of pluripotent cells. Using small molecule antagonists or endogenous inhibitors, it is possible to induce neural conversion to give rise to a population of neural progenitors (Boissart et al., 2013; Chambers et al., 2009; Shi et al., 2012). Neural progenitors may then be patterned into neuronal cell types with regional identities using specific combinations of morphogens, small molecules, growth factors and transcription factors. Depending on the combination and timing of these signals, a variety of neuronal cell types can be obtained, including telecephalic precursors (Watanabe et al., 2005), midbrain dopaminergic neurons (Kawasaki et al., 2000), basal forebrain cholinergic neurons (Bissonnette et al., 2011), spinal motor neurons (Hu and Zhang, 2009), as well as glial cells, such as astrocytes (Serio et al., 2013) and oligodendrocytes (Hu et al., 2009).

It should be noted that protocols for the directed differentiation of hiPSCs into neuronal subtypes are imperfect, often yielding a heterogeneous population of cell types. For instance, derivation of basal forebrain cholinergic neurons from human pluripotent stem cells yields both cells of the basal forebrain cholinergic neuronal lineage as well as interneurons and other cell types (Bissonnette et al., 2011). In addition, each protocol differs in the efficiency of directed differentiation, with some protocols generating highly pure neuronal subtypes (Du et al., 2015), and others achieving lower yields (Kiskinis et al., 2014). Furthermore, many protocols are focused on the directed differentiation of one particular neuronal subtype, neglecting the role of neighbouring cell types, such as glia, that are present in the in vivo environment. In fact, astroglia has been shown to have a critical role in the functional maturation of hiPSC-derived neurons (Tang et al., 2013). Despite these caveats, the last decade of research has seen a vast improvement in neuronal differentiation of human pluripotent stem cells. These efforts have identified novel small molecules that enhance neuronal differentiation and reduce costs, reducing the amount of time required to yield highly enriched populations of specific neuronal subtypes (Kim et al., 2010). In addition, they provide important guidelines and benchmarks for future hiPSC studies of basic neurobiology and disease modelling.

### Using hiPSCs to investigate basic neurobiology

**Fig. 3:**
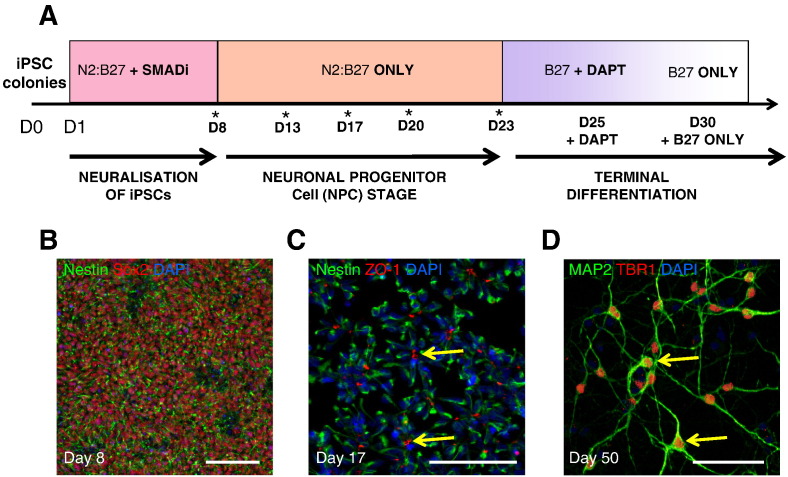
**Corticogenesis in hiPSCs. (A)** Schematic of neuralization protocol: hiPSCs reprogrammed from keratinocytes of healthy individuals were neuralized as a monolayer in the presence of SMAD inhibitors. **(B)** Following 8 days of neuralization, a population of early neuroepithelial cells was formed, as determined by positive staining for nestin and SOX2. **(C)** Subsequent formation of neural progenitor cells (NPCs) was determined by formation of neural rosettes. This stage recapitulates neural tube formation with a clear central pseudo-lumen between concentrically elongated NPCs. Apical lumen of rosettes were positive for ZO-1 with nestin positive cells forming a radial structure surrounding the lumen. **(D)** Terminal differentiation of NPCs, induced by the addition of NOTCH inhibitor DAPT, resulted in the differentiation of projecting glutamatergic neurons positive for MAP2 and TBR1. Scale bar = 100 μm (B + C) and 50 μm (D).

Several studies have recently demonstrated the utility of hiPSCs for functional studies of human neural development. Shi et al. (2012) showed that the distinct steps of human cortical development can be recapitulated in a hiPSC system, from cortical induction to the formation of functional excitatory synapses (Fig. 3). This system closely mirrored the in vivo cortex, in terms of the temporal order of development, specificity of circuit formation, and laminar organisation of projection neurons (Shi et al., 2012). Importantly, this system enabled the generation of all classes of cortical projection neurons for the first time, including the formation of superficial layer neurons, which are absent in mouse cortical development. Similarly, Espuny-Camacho et al. (2013) reported that human corticogenesis can be recapitulated in hiPSCs in the absence of extrinsic morphogens, enabling the generation of diverse pyramidal neurons with distinct patterns of axonal projections and dendritic outgrowths that integrated functionally when grafted in neonatal mouse brain (Espuny-Camacho et al., 2013). These studies show that hESCs and hiPSCs both demonstrate a similar capacity to differentiate into all classes of cortical projection neurons.

GABA interneurons are another major neuronal subtype of the human cortex. Unlike cortical projection neurons, GABA interneurons mainly originate from the medial ganglionic eminence and migrate to the cortex during development (Sussel et al., 1999). Liu et al. (2013a,b) used hiPSCs to examine the development of GABA interneurons. This study showed that the differentiation of GABA interneurons from hiPSCs follows the timeline of human GABA interneuron development, from patterning of primitive neurepithelia to the generation of functional neurons with inhibitory and excitatory inputs (Liu et al., 2013a). Furthermore, multiple GABA interneuron subtypes were observed in this chemically defined system, with specific GABA populations appearing according to their developmental schedule (Liu et al., 2013a). In a related study, this group reported the use of this method to successfully direct the differentiation of hESCs to medial ganglionic eminence progenitors and subsequently, GABA interneurons (Liu et al., 2013b). Interestingly, the transplantation of hESC-derived medial ganglionic eminence progenitors to the hippocampi of mice led to behavioural changes in learning and memory (Liu et al., 2013b). These findings show that in addition to the study of basic neurobiology, hiPSCs and hESCs can be also used to investigate behaviours associated with specific neuronal subtypes.

Similarly, others have investigated the development of other neuronal cell types, including cerebellar Purkinje cells (Wang et al., 2015), retinal cells (Meyer et al., 2009) and motor neurons (Toma et al., 2015). More recently, hiPSC-derived neurons have been used to study specific aspects of cell biology, such as mitochondrial biogenesis (O'Brien et al., 2015) and neuromuscular junction development (Yoshida et al., 2015). These studies show that hiPSC-derived neuronal cell types possess many of cellular and physiological characteristics as hESC-derived and endogenous neuronal cell types. Furthermore, hiPSCs have been used to generate a three-dimensional model of human neural tissue. Lancaster et al. (2013) reported the development of brain tissue from hiPSCs, termed cerebral organoids. Cerebral organoids consisted of discrete regions similar to the cerebral cortex, ventricles and retina tissue, and recapitulated some key aspects of human cortical development mentioned above (Lancaster et al., 2013). Similarly, Meyer et al. (2011) showed that hiPSCs can be differentiated into 3D retinal structures consisting of multiple retinal cell types, in a time frame similar to normal human retinal development (Meyer et al., 2011). These studies provide models to engineer human cortical circuits and investigate cortical function that had not previously been possible with animal models due to species differences.

### Using iPSCs to model and investigate disease

One of the biggest challenges in the study of diseases of the nervous system has been the inaccessibility of live human neural tissue from patients. hiPSC technology combined with methods of directed differentiation provides a general solution to this impediment. An obvious advantage of patient-specific hiPSCs is that they carry the same genetic background as the patient, capturing the mutated genes, as well as the known and unknown genetic modifiers that play important roles in pathogenesis. In addition, patient-specific hiPSCs represent a more physiologically relevant model system, negating the need to overexpress proteins at superphysiological levels in current cellular and animal models. Thus far, a number of groups have published studies of hiPSC neurons from patients with various neurological conditions, including neuropsychiatric disorders schizophrenia (SCZ) (Brennand et al., 2011) and autism spectrum disorders (ASD) (Marchetto et al., 2010; Shcheglovitov et al., 2013), as well as neurodegenerative disorders Alzheimer's disease (AD) (Israel et al., 2012), Parkinson's disease (PD) (Devine et al., 2011) and amyotrophic lateral sclerosis (ALS) (Dimos et al., 2008).

Several groups have reported the generation of hiPSCs from patients with Rett syndrome, an ASD caused by mutations of the *MECP2* gene. Patient-specific hiPSCs maintained the parental mutation and were pluripotent and able to differentiate into the three germ layers (Ananiev et al., 2011; Cheung et al., 2011; Marchetto et al., 2010). All three studies showed that neurons from Rett syndrome hiPSC-derived neurons recapitulated a hallmark feature of ASD, reduction in soma size. In addition, Marchetto et al. (2010) reported that Rett syndrome hiPSC-derived neurons had fewer synapses, reduced spine density and alterations in calcium signalling and defects in electrophysiology.

Altered dendritic arborisation and synaptic density are characteristics that appear to be shared between ASD and SCZ. The generation of hiPSCs from patients with SCZ has also been reported by independent groups. Chiang et al. (Chiang et al., 2011) were the first to generate patient-specific hiPSCs from a patient with mutations in the *DISC1* gene, albeit without reporting any SCZ-relevant phenotypes. Soon after, Brennand et al. (2011) reported the generation of hiPSCs from patients with childhood-onset SCZ or from families affected with psychiatric disease, likely reflecting a genetic component to disease. Patient-specific hiPSCs were indistinguishable from control hiPSCs in pluripotency and in their ability to differentiate into functional neurons. However, patient-specific hiPSC-derived neurons displayed decreased neuronal connectivity, reduced neurite outgrowth and decreased PSD95 protein levels, despite normal spontaneous neuronal activity (Brennand et al., 2011). Interestingly, a more recent study on hiPSCs generated from patients with a family history of SCZ reported a discrepancy in these observations. Robicsek et al. (2013) reported that patient-specific hiPSC-derived neural progenitor cells (NPCs) exhibited abnormal morphology and a delay in differentiation into dopaminergic neurons and an inability to differentiate into glutamatergic neurons (Robicsek et al., 2013). hiPSCs have also been generated from 22q13 deletion syndrome patients (Phelan-McDermid syndrome), who display intellectual disability and an increase risk of ASDs. Amongst the gene deleted in this region, *SHANK3*, which encodes a post-synaptic scaffold protein, has also been associated with ASD, intellectual disability and SCZ. Neurons derived from these patient-specific hiPSCs demonstrated reduced levels of Shank3 expression and displayed deficits in excitatory transmission and synaptic number (Shcheglovitov et al., 2013). Remarkably, the authors further demonstrated that overexpression of Shank3 or treatment with IGF1 was sufficient to restore synaptic deficits in these patient-specific hiPSC-derived neurons (Shcheglovitov et al., 2013). Collectively, these studies have provided proof of concept by demonstrating that cells derived from patient-specific hiPSC are a cellular system in which to confirm and elucidate the impact of genetic variations associated with complex disorder on cellular phenotypes. However, more recently, several studies have now used patient-specific hiPSCs as a tool to investigate novel mechanisms and cellular phenotypes that may contribute to the pathophysiology of neurodevelopmental disorders. For example, Wen et al. (2014) generated hiPSCs from 4 family members 2 of which had a frameshift mutation in the *DISC1* gene and were diagnosed with SCZ or major depression, and 2 unaffected family members with the mutation. Neurons generated from hiPSC of the affected family members displayed reduced levels of the DISC1 protein, abnormal synaptic transmission and reduced synapse number, consistent with previous reports using animal models (Hayashi-Takagi et al., 2010; Wen et al., 2014). However, the authors further discovered that reduction in DISC1 expression also resulted in the dysregulation of multiple genes related to synaptic and psychiatric disorder, hence uncovering a novel transcriptional regulatory action for the DISC1 protein (Wen et al., 2014). Around the same time, Yoon et al. (2014) generated hiPSCs from SCZ patients with a 15q11.2 microdeletion; one of the genes expressed in this region is *CYFIP1*, which has been associated with SCZ and ASDs. Using NPCs derived from these patient-specific hiPSCs, the authors identified that haploinsufficiency of *CFYIP1* resulted in a disruption in adherent junctions and apical polarity, an effect that they further demonstrated to disrupt radial glial cell localization in a mouse model (Yoon et al., 2014). This study further demonstrates how hiPSCs can be an entry point to discovering novel insights into disease pathology (Durak and Tsai, 2014).

In addition to these studies, hiPSCs have also been used to successfully model aspects of late-onset neurodegenerative disorders. Shortly after the generation of the first hiPSCs, Dimos et al. (2008) were the first to report the generation of patient-specific hiPSCs from an 82-year-old woman with ALS, and their differentiation into motor neurons. Although this study did not report any disease phenotypes, a number of studies have since shown that patient-derived hiPSCs do recapitulate disease-relevant phenotypes upon neuronal differentiation. After the initial proof-of-principle study demonstrating the feasibility of generating patient-specific hiPSCs and their differentiation into motor neurons, several groups reported the generation of hiPSCs from ALS patients with known and well characterised pathogenic mutations. These studies have shown that neurons and glia derived from patient-specific hiPSCs recapitulate key pathological phenotypes of ALS, including mutant cellular and biochemical features, and cellular vulnerability (Almeida et al., 2013; Bilican et al., 2012; Egawa et al., 2012; Sareen et al., 2013). A comprehensive study of hiPSCs generated from patients with familial AD and sporadic AD reported key features of AD, such as higher levels of amyloid-beta and phosphorylated tau, in both familial and sporadic AD hiPSC-derived neurons (Israel et al., 2012). Similarly, midbrain dopaminergic neurons from hiPSCs generated from a familial PD patient with a mutation in the gene encoding alpha-synuclein exhibit increased levels of alpha-synuclein protein, recapitulating the cause of PD (Devine et al., 2011). Other PD-relevant phenotypes have also been observed in hiPSC-derived neurons from PD patients with mutations in the *PINK1* and *LRRK2* genes, including increased production of mitochondrial reactive oxygen species and abnormal mitochondrial transport (Cooper et al., 2012).

Several recent studies have also combined genome editing tools with hiPSCs to both model the cellular phenotypes associated with ALS, the most common adult-onset motor neuron disease, and to investigate the genetic contribution to disease pathogenesis. Two independent groups reported the generation of hiPSCs from ALS patients with mutations in the *SOD1* gene (Chen et al., 2014; Kiskinis et al., 2014). Kiskinis et al. (2014) reported that patient-specific hiPSC-derived motor neurons exhibited increased cell death and reduced soma size and shorter neurites, whereas Chen et al. (2014) observed aggregation of mutant SOD1 protein and neurofilament inclusions in patient-specific hiPSC-derived motor neurons (Chen et al., 2014; Kiskinis et al., 2014). Kiskinis et al. (2014) and Chen et al. (2014) used zinc-finger nuclease and TALENs, respectively, to target the correction of the *SOD1* mutation in the patient-specific hiPSCs. Both studies showed that genetic correction of the *SOD1* mutation rescued the ALS-associated phenotypes.

These studies provide support for the use of hiPSCs for modelling the molecular pathogenesis of diseases of the nervous system. In addition to disease modelling, patient-specific iPSCs may be used to investigate disease mechanisms that may not be exposed in non-neuronal cell types. As iPSC technology and protocols for directed differentiation continues to develop, hiPSC models have the potential to greatly reduce the time and costs associated with clinical trials of drug discovery.

## Limitations of iPSCs

### Technical limitations

Despite the tremendous potential of hiPSCs, a number of technical limitations currently restrict hiPSC-based studies. These limitations relate primarily to several forms of variability arising from differences between hiPSC-neurons derived from a single patient, from independent clones derived from a single patient and from differences between hiPSC lines derived from different patients. In order to address these constraints, large experiments consisting of multiple neuronal differentiations from multiple independent hiPSC lines from multiple patients would have to be performed. Due to the time constraints and costs involved with these large experiments, most hiPSC studies have used a minimal number of cell lines and neuronal differentiations for proof-of-principle experiments.

Most established methods of directed differentiation result in heterogeneous populations of differentiated cells. These impure populations typically consist of different subtypes of neurons, as well as non-neuronal cell types. This heterogeneity leads to differences between individual hiPSC neurons derived from a single patient, or neuron-to-neuron variability. Some studies have addressed this issue by using fluorescent-activated cell sorting (FACS) to purify specific neuronal subtypes. To do this, hiPSC-derived neuronal cultures may be transfected or transduced with a plasmid or a virus encoding a fluorescent protein under the control of a subtype-specific promoter, such that only the specific neuronal subtypes will express the fluorescent protein, which can subsequently be sorted from the heterogeneous population. This has been used to identify small molecules that improve survival of hiPSC motor neurons from ALS patients (Yang et al., 2013). Although FACS generates highly pure cultures and minimises neuron-to-neuron variability, this method carries a risk of contamination and also results in a lower yield of cells.

In addition to this, hiPSC clones derived from a single patient are known to exhibit differences genetically and in differentiation propensities (Hu et al., 2010). Genetic variability may arise from the reprogramming process, due to differences in the number of viral integrations or spontaneous mutations (Gore et al., 2011). While the former issue may be addressed by non-integrating viral vectors or the use of small molecules, spontaneous mutations occur naturally and are an inherent variability in hiPSC-based studies. Indeed, a major limitation of hiPSC research stems from the accumulation of mutations in rapidly cycling cultured cells, such as hiPSCs and human ESCs (Baker et al., 2007; Mayshar et al., 2010). Duplication of chromosomal regions has been reported to occur in a proportion of the hiPSC and human ESC lines analysed (Taapken et al., 2011). Biased gene expression associated with such chromosomal aberrations has also been observed in some of the hiPSC lines analysed, as a result of culture adaptation, although biased gene expression was also found at an early passage of one hiPSC line (Mayshar et al., 2010). To work around these limitations, studies may be performed on early passage hiPSC clones that have passed rigorous quality controls, including morphological, gene expression and karyotype analyses, tests for contaminants, and with RNA and DNA methylation profiling.

Another form of variability derives from differences between hiPSC lines derived from different patients, or inter-patient variability. For instance, hiPSC lines have been shown to differentiate into neuronal lineages with variability in differentiation efficiency and in electrophysiological properties (Hu et al., 2010). Inter-patient variability has also been associated with donor identity and sex (Boulting et al., 2011). Some studies have taken advantage of the recent development of genome editing technologies to circumvent such variability by generating isogenic hiPSC lines. Much of these differences continue to reduce with the development of pluripotent stem cell technology. The use of commercially available cell culture media, substrates and reprogramming kits has increased the quality and consistency of hiPSC cultures. The development of protocols for the directed differentiation of hiPSCs has led to better defined culture conditions and more reliable neuronal cultures.

### Reproducibility

In addition to the intra- and inter-patient variability discussed above, it is important to consider the reproducibility of hiPSC-based studies. Unlike studies involving the use of well-established cell lines, the experimental design in iPSC-based studies is heavily debated. For instance, it is not clear how many patients or iPSC lines should be included in a study, nor is it clear whether data from different studies can be compared, due to differences in the origin of the donor tissue, reprogramming method or differentiation protocol.

It has been suggested that iPSCs retain a memory of the tissue from which they are derived, which could affect their ability to differentiate into certain cell lineages (Marchetto et al., 2009). Subtle differences in global gene expression have been reported between iPSCs derived from different somatic tissues from the same individual, and between iPSCs derived the same individual, but reprogrammed by different methods (Rouhani et al., 2014). Despite this, these differences are considerably less than that observed for inter-individual transcriptional variation in iPSCs (Rouhani et al., 2014). Therefore, it is likely that cellular phenotypes between different iPSC lines are likely driven by different genetic backgrounds rather than donor tissue or reprogramming method. Indeed, an examination of multiple iPSC lines derived from different individuals showed greater inter-individual than intra-individual differences in expression of motor neuron markers (Boulting et al., 2011). These findings suggest that iPSC-based studies should focus on working with iPSC lines from different donors rather than multiple lines derived from the same individual (Rouhani et al., 2014).

The issue regarding the use of differentiation protocols remains unclear. Similar differentiation efficiencies have been obtained by independent laboratories using the same standardised procedures (Boulting et al., 2011), supporting the reproducibility of iPSC-based studies. However, many iPSC-based studies use alternative differentiation protocols, which make it difficult to interpret findings from different studies. Two independent studies using different differentiation protocols have reported an identical biochemical phenotype in iPSC-derived neurons with the same genotype (Bilican et al., 2012; Egawa et al., 2012). Alternatively, two independent studies using different differentiation protocols recently reported dissimilar rates of cell death in iPSC-derived neurons with the same genotype (Chen et al., 2014; Kiskinis et al., 2014). As mentioned previously, the development of simpler and more affordable methods for hiPSC culture and differentiation should enable the generation of robust, large-scale neuronal cultures to enable reproducibility of hiPSC studies.

### Time

A notable constraint in the use of iPSCs for studies of basic and disease mechanisms regards the time frame of in vitro experiments and the time required for the onset of psychiatric and neurological diseases. It has been shown that the gene expression profiles of hiPSC-derived neural progenitors shared highly similar gene expression profiles with brain tissue at 8–16 weeks post conception, whereas hiPSC-derived neurons shared the most similarity gene expression profiles with brain tissue at 8–24 weeks post conception (Brennand et al., 2015). These findings present a challenge for the study of adult-onset neurological conditions. Indeed, most reports of adult-onset disorders or late-onset neurodegenerative conditions have not been able to model the loss of neurons that is typical in human disease. Rather, these studies have identified susceptibility to particular cellular stressors (Bilican et al., 2012; Israel et al., 2012), instead of explicit degeneration.

A recent study showed that it may be possible to circumvent the fetal identity of iPSC-derived neurons. Progerin is a truncated form of a nuclear membrane protein and is involved in Hutchinson–Gilford progeria syndrome, a genetic disorder characterised by premature ageing. Miller et al. (2013) found that the expression of progerin protein leads to the induction of ageing-related events in hiPSCs. Importantly, the expression of progerin in hiPSCs from Parkinson's disease patients enabled the emergence of disease-relevant phenotypes (Brennand, 2013; Miller et al., 2013). Although the expression of progerin appears to accelerate the maturation of iPSC-derived neurons, it likely does not reflect all aspects of ageing and alternative approaches need to be developed to address the issue of age identity.

## Modelling the effects of estrogens in human stem cells and iPSCs

### Estrogens and estrogen receptors in human stem cells

To date there have been a select few studies that have investigated the effects of estrogens in human neural stem cells (hNSCs) or neural progenitor cells (hNPCs). In this review we define NSCs and NPCs as any self-renewing neural cell capable of differentiation into neurons, astrocytes and/or oligodendrocytes, and will use these terms interchangeably. The study by Hong et al. (2004), was one of the first to investigate the expression of ERs and other steroid receptors in human embryonic stem cells (hESCs), multipotent cells capable of differentiating into various cell types from the 3 germ layers (Hong et al., 2004). This study demonstrated that mRNA for ERα, ERβ, glucocorticoid receptor (GR), and progesterone receptor (PR) were present in hESCs, but did not investigate ER expression beyond this stage (Hong et al., 2004). However, it was not until Kishi et al. (2005) that the presence of these receptors and the effect of 17β-estradiol on hNSC differentiation were demonstrated. In this study, both ERα and ERβ were found to be expressed in hNSCs derived from fetal mesencephalic tissue. Differentiation of these midbrain hNSCs further gave rise to a population of tyrosine hydroxlase positive neurons in which a similar proportion of these neurons expressed ERα and ERβ as determined by immunofluorescence (Kishi et al., 2005). Interestingly, 17β-estradiol increased the number of tyrosine hydroxlase positive neurons following differentiation in vitro, in a dose-dependent manner, and moreover, in vivo after transplantation into mouse brains. Thus, these data indicated that 17β-estradiol could influence the differentiation of mesencephalic hNSC into dopamine neurons in vitro and in vivo. It should be noted that the effect of 17β-estradiol on the number of grafted hNSCs in vivo could be due to an increase in cell survival following transplantation (Kishi et al., 2005).

In addition to regulating differentiation, 17β-estradiol has also been shown to influence the proliferation of hNPCs. Using hNPCs derived from fetal cortex, Wang et al. (2008) showed that treatment with 17β-estradiol increased hNSC proliferation in a dose- and time-dependent manner. Assessment of ER expression revealed a predominate expression of ERβ; ERα expressed was reported to be barely detectable (Wang et al., 2008). Using ER selective agonists, the authors further demonstrated that the ERβ agonist, DPN, but not the ERα agonist, PPT, was capable of increasing hNPC proliferation. Treatment with 17β-estradiol and DPN, but not PPT, also resulted in an increase in phosphorylation of the MAP kinases, ERK1/2. Critically, 17β-estradiol and DPN induced cell proliferation was blocked in the presence of the MEK kinase (a direct regulator of ERK1/2 phosphorylation) inhibitor, U0126 (Wang et al., 2008). Therefore, the data from this study indicates that ERβ is the predominate ER in cortical hNPCs, and mediates hNPC proliferation via a MEK/ERK1/2-dependent pathway.

Several studies have also investigated ER expression, and the function of 17β-estradiol in human neurons derived from either fetal tissue or hESCs. Fried et al. (2004) demonstrated that ERβ was highly expressed in primary cultures of neurons and glial cells generated from human embryo cortex and spinal cord. Furthermore, treatment with 17β-estradiol increased the expression of ERβ (Fried et al., 2004). While this study suggested that young developing human neurons do express ERβ, it was not clear whether ERα is also expressed in fetal neurons. More recently, Zhang et al. (2010) have shown that ERβ, but not ERα is expressed in neurons differentiated from the hESC lines, H7 and H9. In order to determine whether 17β-estradiol, or activation of either ERα or ERβ could influence cell function, the authors examined Ca^2 +^ oscillations (Zhang et al., 2010). In both H7 and H9-derived neurons, 17β-estradiol rapidly (within minutes) increased the frequency, amplitude and synchrony of Ca^2 +^ oscillations. Additionally, treatment of these neurons with 3 independent ERβ agonists, DPN, WAY-202041 or MF101, also increase the frequency, amplitude and synchrony of Ca^2 +^ oscillations within a few minutes (Zhang et al., 2010). However, the ERα agonist, PPT, had no effect on Ca^2 +^ oscillations, mirroring the apparent lack of ERα expression in H7 or H9-derived neurons. Similar results were also obtained in neurons derived from a mouse ESC line. Taken together, the data from these studies suggest that ERβ can not only regulate hNPC proliferation, but is also able to regulate Ca^2 +^ signalling in human neurons derived from an hESC source, and thus may play a predominate role during neuronal development.

These studies are amongst the first to study the effects of 17β-estradiol on cellular physiology in human neural tissue. However, a number of limitations are associated with hNSC and hESCs. Firstly, a considerable amount of variation is seen between different cell lines (Adewumi et al., 2007), making it difficult to generalise and reproduce results across different lines. In addition, NSCs only differentiate into specific neural lineage, determined by which brain region they were a derived from. Critically, hNSCs and hESCs do not offer the ability to recapitulate the ploygenic background associated with specific neurological diseases. Nevertheless, these studies do offer us important insights into the actions of estrogens in human neurons. For example, a number of animal studies, both in vivo and in vitro, have reported similar effects of 17β-estradiol to that observed in hNSCs and hESCs. Treatment of rat glioma and mouse hippocampal neurons with 17β-estradiol has been shown to improve cell viability (Behl et al., 1995; Bishop and Simpkins, 1994), and administration of 17β-estradiol was shown to reduce mortality from focal ischemia in rats (Zhang et al., 1998). In addition, increases in dendritic spine density following exposure to estrogens have been observed in primary hippocampal neurons (Murphy and Segal, 1996), cortical neurons (Srivastava et al., 2008) and in cortical and hippocampal neurons of adult rats (Inagaki et al., 2012; Woolley and McEwen, 1992). Moreover, Zhang et al. (2010) directly compared the effect of 17β-estradiol on Ca^2 +^ oscillations in neurons derived from hESCs and mouse ESCs, and reported similar increases in Ca^2 +^ signalling and activation of kinase pathways in neurons from human or mouse ESCs suggesting a common mechanism of action. Thus, these findings provide confidence that in vitro experiments using hNSC/ESCs or hiPSC neurons would be able to model the actions of 17β-estradiol within the human brain.

### Characterising the effects of 17β-estradiol in hiPSCs

To our knowledge, no study has thus far investigated whether estrogens are functionally active in neurons derived from hiPSCs. To this end we established an iPSC line from hair keratinocytes of a healthy male individual. Hair keratinocytes were reprogrammed using nonintegrating approach; cells were transduced with Sendai virus expressing OCT4, SOX2, KLF4 and C-MYC (kind gift of M. Nakanishi, AIST Japan) (Nishimura et al., 2011; Takahashi et al., 2007a) (also see Cocks et al. (2014)). Detailed quality control analyses of the hiPSC line was performed as previously described (Cocks et al., 2014). In order to investigate the effects of 17β-estradiol in neurons differentiated from this hiPSC line, we utilized a neuralization protocol that has previously been used to generate forebrain/cortical neurons (Cocks et al., 2014; Shi et al., 2012). Briefly, hiPSCs were differentiated in a monolayer, in the presence of SMAD inhibitors, Dorsomorphin, and SB431542 (Fig. 3A). This resulted in the generation of a relatively homogenous population of neuroepithelial cells, which were positive for the NSC markers, nestin, an intermediate filament protein, and SOX2, a marker of self-renewal, or pluripotency (Fig. 3B) (Chambers et al., 2009; Cocks et al., 2014; Shi et al., 2012; Yoon et al., 2014). Subsequent passaging of this population of neuroepithelial cells resulted in the generation of cortical neural rosettes (Fig. 3C) (Chambers et al., 2009; Cocks et al., 2014; Shi et al., 2012; Yoon et al., 2014). The apical lumen of neural rosettes showed robust expression of the adhesion marker, ZO-1, representing the typical formation of apical–basal polarity in hNPCs (Fig. 3C) (Cocks et al., 2014; Shi et al., 2012). Terminal differentiation of hNPCs into neurons was achieved by the addition of the NOTCH inhibitor DAPT for 5 days (Cocks et al., 2014). This gave rise to the generation of a large number of TBR1-positive neurons, a marker of layer V cortical neurons (Espuny-Camacho et al., 2013; Shi et al., 2012), indicating that the majority of cells had differentiated into excitatory forebrain/cortical projection neurons (Fig. 3D).

While the forebrain consists of both glutamatergic projection neurons and GABAergic interneurons, previous studies have shown that forebrain excitatory glutamatergic neurons are generated by cortical progenitor cells, whereas forebrain GABAergic interneurons originate from the ganglionic eminence and migrate into the cerebral cortex (Sussel et al., 1999). Several recent studies have shown that the differentiation of hiPSCs into these two neuronal subtypes requires different inductive signals. Shi et al. (2012) reported that the combination of retinoid signalling and SMAD inhibition led to the induction of neuroepithelia with apico-basal polarity unique to cortical progenitors, which subsequently differentiated into cortical projection neurons. In contrast, the differentiation of GABAergic interneurons requires the ventralisation of primitive neuroepithelia into medial gangolionic eminence-like progenitors, which subsequently differentiate into various GABAergic interneuron subtypes (Liu et al., 2013a). In our lab, we have focused on the generation of glutamatergic projection neurons, which represents the majority of neurons in the cerebral cortex. However, the study of GABAergic interneurons, and the co-culture of these two neuronal subtypes, is warranted to better recapitulate cortical development.

**Fig. 4:**
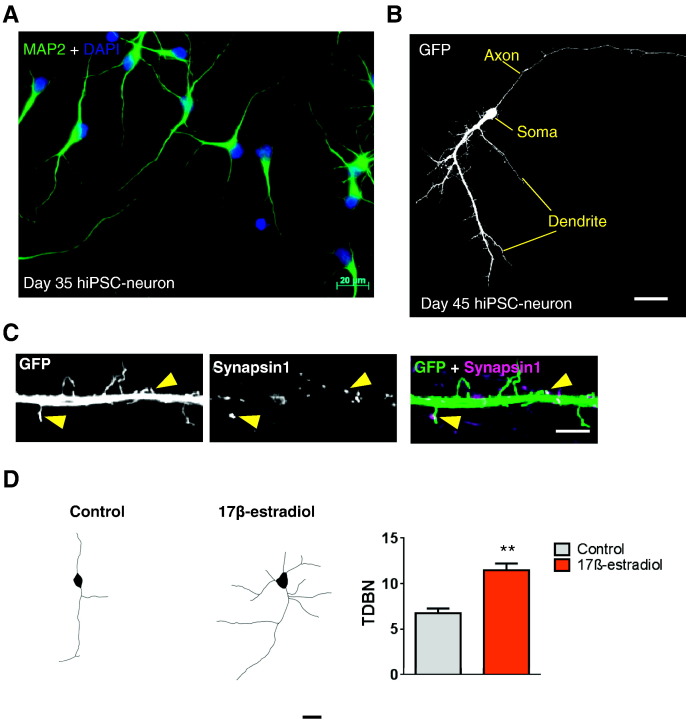
**Differentiated hiPSC-neurons display features of pyramidal neurons. (A)** Representative image of hiPSC-neurons differentiated for 35 days and stained for MAP2 (green) with DAPI nuclear stain (blue). hiPSC-neurons display the development of neuronal morphology with dendrites clearly present. Additional smaller processes protruding from the cell soma or from main dendrites are also evident, demonstrating a limited level of arborisation. **(B)** Expression of eGFP in 45 hiPSC-neurons differentiated for 45 days. By this age, hiPSC-neurons display a polarised morphology; an axonal process can be identified, and a single, primary dendrite with secondary and tertiary branches is also evident. **(C)** High magnification images of dendrite of day 45 hiPSC-neurons expressing eGFP and co-stained for the pre-synaptic marker. This reveals the presence of filipodia and immature (thin) dendritic spines. Occasionally, immature dendritic spines co-localize with synapsin1 (yellow arrow heads) suggesting that these cells are undergoing synaptogenesis. **(D)** Treatment with 17β-estradiol (10 nM) for 24 h results in an increase in dendritic branching. N = 18–23 cells per condition, from 3 experiments. **p < 0.01, Student *t*-test. Scale bar, 20 μm **(A + B)**, 5 μm **(C),** 10 μm **(D)**.

An important characteristic of cortical glutamatergic neurons is their ability to generate unipolar pyramidal neuronal morphology; interneurons develop multipolar morphologies (Dotti et al., 1988; Gaspard et al., 2008; Markram et al., 2004). Previously it has been shown that cortical neurons differentiated from hESCs also generate unipolar pyramidal neuronal morphologies (Gaspard et al., 2008). After 35 days of differentiation, we found that the majority of MAP2-positive cells displayed a unipolar morphology that would be associated with a young developing neuron (Fig. 4A). In order to examine the morphology of neurons differentiated for longer periods of time, we transfected cells with eGFP to outline cell morphology. By day 45 hiPSC neurons had adopted a polarised neuronal morphology; Fig. 4B shows a representative image of an eGFP expressing hiPSC-neuron that has formed a single primary dendrite with secondary and tertiary dendritic arborisations, and the characteristic specification of an axonal process (Fig. 4B). This morphology is indicative of a young, yet maturing pyramidal neuron. During early postnatal development, synaptic dendritic protrusions first appear as long, thin, highly motile structures known as filopodia, which can initiate synaptic contacts with nearby axons (Yoshihara et al., 2009; Ziv and Smith, 1996). This initial contact between pre- and post-synaptic sides is a key step in synaptogenesis. The subsequent maturation and stabilisation of synapses are thought to involve filipodia transforming into highly specialised dendritic protrusions known as dendritic spines (Yoshihara et al., 2009; Ziv and Smith, 1996). Consistent with this hiPSC-neurons grown for 45 days also displayed dendritic protrusions along the dendritic shaft (Fig. 4C). Interestingly, a number of these dendritic protrusions co-localized with the pre-synaptic marker, synapsin1, suggesting that these protrusion maybe very immature dendritic spine involved in synaptogenesis (Fig. 4C).

Following the establishment of hiPSC neurons with a forebrain/cortical lineage, we next asked whether these cells could respond to the application of 17β-estradiol. Thus, we took advantage of the powerful effect that 17β-estradiol has on regulating neuronal morphology during development (Arevalo et al., 2012; Srivastava and Evans, 2013; Srivastava et al., 2013). To this end, we treated day 34 hiPSC-neurons with either 17β-estradiol (10 nM) for 24 h. Examination of neuronal morphology of MAP2-positive day 35 neurons revealed that 17β-estradiol treatment increased the number of dendritic branches (Fig. 4D), an observation consistent with previous studies (Arevalo et al., 2012). While this indicates that hiPSC-neurons are indeed responsive to 17β-estradiol, it also demonstrates that male human neurons are responsive to estrogenic signalling. These preliminary observations provide evidence that 17β-estradiol is capable of inducing structural changes in neurons differentiated from hiPSCs, derived from a healthy male individual. It will be critical in the future to confirm that these neurons are indeed functional, and to investigate the expression of ERs in these cells. Nevertheless, these data indicate that hiPSCs are a suitable platform from which to investigate the role of estrogens during neuronal development and even in disease.

## Conclusions

In this review we have attempted to highlight the recent advances in the field of stem cell research and in particular iPSCs. It is clear that this field of study rapidly developing and moreover, that this approach does hold much potential for investigating the basic mechanisms of neural developments (Gaspard and Vanderhaeghen, 2011). Critically, this approach is emerging as a key tool for further developing our understanding of neurodevelopmental and degenerative disease by revealing novel insight into disease pathophysiology or the screening of potential therapeutic compounds (Brennand et al., 2012; Cocks et al., 2014; Dolmetsch and Geschwind, 2011; Durak and Tsai, 2014; Gaspard and Vanderhaeghen, 2011; Yu et al., 2013). A major advantage of hiPSCs over hNSCs or hESCs, is that they can be generated using readily accessible tissue from individuals of any age and they can be combined with methods of directed differentiation to enable accessibility to multiple different neural cell types. Human NSCs and ESC are derived from fetal tissue, and thus are much harder to access. It is also unclear how much variability there are between hNSCs and hESCs, and there is considerable heterogeneity in their ability to differentiate into neural tissue (Adewumi et al., 2007). Moreover, neurons generated from NSC or ESCs are fate restricted, that is they are only capable of generating neural cells of a specific lineage (Adewumi et al., 2007; Gaspard and Vanderhaeghen, 2011). Critically, generation of iPSCs from patients diagnosed with specific diseases, means that it is possible to model the disorder using neurons that faithfully recapitulate the cellular environment of a disease state. However, iPSC technology is still in its infancy with several limitations, although this aspect of the technology is currently under being research by many laboratories. Moreover, this in vitro technology is unlikely to replace animal models of disease, but as has already been shown, provides a complementary tool, and an entry point in identifying novel pathogenic mechanisms that can subsequently be modelled in vivo using animal models. Despite these caveats and limitations, several advances in our understanding of neurological disorders have already been made using patient-derived hiPSCs (Brennand et al., 2012; Durak and Tsai, 2014; Gaspard and Vanderhaeghen, 2011).

As stated above, estrogens have a powerful influence over cognition. Moreover, estrogens or estrogen-based therapies continue to be suggested as potential therapeutic strategies, despite well described side effects. We propose that one route to understanding the full potential of such strategies and in order to develop more effective and safer estrogen-based therapies, is to utilize the ability of hiPSCs to provide a native human cellular environment. This enables us to investigate the actions of estrogens at a molecular level in native human neurons. Moreover, it would allow for a greater understanding of how estrogens may affect cellular processes in a physiologically relevant cellular model carrying the precise genetic variants that contributed to neurological conditions. As our preliminary data indicates that hiPSCs are indeed responsive to 17β-estradiol, we feel that this is an achievable idea, although, this work needs to be carefully replicated and considered in light of the limitations as an in vitro system. Patient-derived hiPSCs that recapitulate the complex genetics associated with many disorders of the brain, have the potential to fill a critical gap in determining both how estrogens, or altered levels of estrogens, may contribute to pathogenetic mechanism, and moreover, provide platform from which to develop more effective and safer estrogen-based therapeutics, even in a sex-specific manner.
